# Personalized Treatment Selection for Digital Eating Disorder Interventions: A Proof‐of‐Concept for the Personalized Advantage Index

**DOI:** 10.1002/eat.70112

**Published:** 2026-04-21

**Authors:** Zoe McClure, Matthew Fuller‐Tyszkiewicz, Mariel Messer, Jake Linardon

**Affiliations:** ^1^ School of Psychology, Deakin University Geelong Victoria Australia

**Keywords:** digital intervention, eating disorder, personalized advantage index, treatment selection

## Abstract

**Objective:**

Although a range of evidence‐based treatments for eating disorders exist, treatment response varies substantially. The ability to match individuals to a treatment which they are most likely to benefit from may help improve treatment efficiency and therapeutic outcomes. The present study applies a treatment selection approach called the personalized advantage index (PAI) and evaluates its utility for matching individuals to a broad versus focused digital program for eating disorder symptoms.

**Method:**

Data were used from a randomized non‐inferiority trial comparing the two interventions (*n* = 214). Machine learning models (elastic net and random forest) were trained to predict post‐intervention symptom severity for a broad and focused digital intervention using 40 self‐reported baseline predictors. The PAI was calculated to identify the predicted optimal treatment for each participant.

**Results:**

Elastic net performed marginally better than the random forest at predicting intervention outcomes (Elastic net *R* ^2^ = 0.29; Random Forest *R* ^2^ = 0.26). Independent samples *t*‐tests indicated no significant differences in post‐intervention outcomes between participants who received their PAI‐indicated treatment and those who did not in both the full sample and a subsample with larger predicted differential responses.

**Discussion:**

PAI‐based treatment matching did not improve post‐treatment outcomes in this context. Greater utility of the PAI approach may emerge when applied to different treatment orientations or delivery formats, enabling greater opportunity for differential effects to emerge.

## Summary

- We investigated the utility of the personalized advantage index (PAI) for matching participants to a broad versus focused digital program for eating disorder symptoms.
- The PAI was derived by training machine learning models to predict individual‐level responses to each intervention using baseline predictors.
- Participants who had received their PAI‐recommended intervention did not have significantly better outcome at post‐intervention compared to those who did not receive their PAI‐recommended intervention.
- Enhanced utility may require identification of more robust intervention moderators and application to interventions which differ more substantially in therapeutic approach or modality.

## 1 Introduction

Cognitive behavioral therapy (CBT) is a first‐line treatment for eating disorders characterized by recurrent binge eating, with strong empirical support across diagnoses (Cuijpers et al. 2025; Grilo 2024). The enhanced transdiagnostic form of CBT (CBT‐E) is structured and modular, typically beginning with psychoeducation and regular eating, followed by targeted strategies addressing key maintaining mechanisms such as dietary restraint, systematic problem solving, exposure to feared foods, body image disturbance, mood intolerance, and, where indicated, supplementary modules targeting perfectionism, interpersonal difficulties, and core low self‐esteem (Fairburn 2008). Although CBT‐based treatments reliably reduce symptoms at the group level, substantial variability in individual response is common. Some individuals experience marked improvement, others show limited change, and a subset deteriorate (Cuijpers et al. 2025; Messer et al. 2025). This heterogeneity highlights the need for approaches that move beyond average treatment effects and instead identify which individuals are most likely to benefit from specific therapeutic components or delivery formats.

One promising approach for improving treatment selection is the personalized advantage index (PAI), which estimates how much better (or worse) an individual is expected to respond to one treatment compared with an alternative (DeRubeis et al. 2014). The PAI is derived by fitting multivariable prediction models that estimate individual‐level outcomes under each treatment condition based on baseline characteristics, and then comparing these predicted outcomes to determine which treatment is expected to yield the superior result for a given person (DeRubeis et al. 2014; Meinke et al. 2024). PAI models have been generated using both traditional multivariable linear regression approaches and more complex machine learning algorithms capable of modeling non‐linear relationships and higher‐order interactions (Meinke et al. 2024). By translating multivariable prediction models into a single, clinically interpretable metric, the PAI offers a structured, data‐driven method for guiding treatment selection (Cohen and DeRubeis 2018). In principle, this approach may reduce reliance on trial‐and‐error decision making and improve the efficiency of care allocation.

The utility of the PAI approach has been evaluated across several mental health conditions, with promising results. Studies comparing treatment options for depression, post‐traumatic stress disorder (PTSD), and somatic symptom disorder have found significantly better outcomes among those who received their PAI‐indicated treatment compared to those who did not, particularly when the predicted difference in treatment response was large (Bauer‐Staeb et al. 2023; Delgadillo and Gonzalez Salas Duhne 2020; DeRubeis et al. 2014; Huibers et al. 2015; Lopez‐Gomez et al. 2019; Wade et al. 2025). Notably, most PAI investigations have compared treatment options that differ in therapeutic orientation (e.g., CBT versus IPT) or treatment modality (e.g., psychotherapy versus pharmacotherapy) using self‐reported or interview‐based sociodemographic and clinical data as input. For example, in the first application of the PAI, DeRubeis et al. (2014) developed a generalized linear model to match individuals with depression to either antidepressant medication or CBT using five identified moderators of treatment response (marital status, employment status, life events, comorbid personality disorder, and prior medication trials). Among participants for whom a clinically significant treatment advantage was predicted (60% of the sample), those who received their predicted optimal treatment had significantly lower symptom severity at post‐intervention compared to those who received a non‐optimal assignment (DeRubeis et al. 2014).

A small number of studies have applied the PAI to variations within the same therapeutic framework. For example, Friedl et al. (2020) used Bayesian model averaging and linear regression to match individuals with depression to standard CBT versus an enhanced CBT condition incorporating additional exposure and emotion‐focused elements. Thirteen sociodemographic and clinical variables were included as predictors. For 46% of participants, the model predicted a clinically meaningful difference in post‐treatment outcome, defined as a five‐point difference on the BDI‐II, between optimal and non‐optimal treatment assignment. Similarly, Held et al. (2023) developed a PAI to match individuals with PTSD to either a three‐week or two‐week intensive CBT‐based program. Although most participants were predicted to benefit similarly from either format, a small subgroup, 12%, were expected to experience a clinically meaningful advantage if assigned to their PAI‐indicated treatment. Together, these studies suggest that even within a shared therapeutic orientation, differences in structure, intensity, or targeted mechanisms may produce meaningful individual‐level variation in response. This provides a conceptual basis for examining whether data‐driven matching approaches can improve outcomes when comparing related interventions. However, the utility of the PAI for guiding treatment selection in eating disorders has not yet been evaluated.

This proof‐of‐concept study aimed to evaluate the utility of the PAI approach for matching individuals to a broad versus focused digital intervention for eating disorder symptoms. We used data from a recent RCT that compared the effectiveness of these two digital interventions (Linardon et al. 2023). Both interventions are grounded in CBT but differ in scope, targeted change mechanisms, and delivery format (app versus web‐based). The broad program targeted multiple maintaining mechanisms, including dietary restraint, mood dysregulation, and body image concerns, whereas the focused program specifically targeted dietary restraint. Although equivalent effects were observed at the group level, substantial individual variability was evident in treatment response, suggesting the possibility of meaningful heterogeneity that may not be detectable through traditional group‐level analyses (Linardon et al. 2023).

Importantly, although robust treatment moderators have not yet been established in digital eating disorder interventions (McClure et al., 2023), prior research in this area has typically examined a relatively narrow set of demographic and clinical predictors. In contrast, the present trial collected a comprehensive range of baseline characteristics, including sociodemographic, clinical, motivational, attitudinal, and technology acceptance variables, many of which have not previously been evaluated as potential prescriptive factors in this context. The breadth and theoretical relevance of these variables provided a strong empirical basis to test whether more sophisticated modeling approaches could identify differential response patterns that traditional analyses may overlook. Given increasing enthusiasm for digital eating disorder programs (Torous et al. 2025), determining whether individuals can be matched to the intervention most suited to their characteristics represents an important and timely question. Even if differential matching effects prove modest, empirically evaluating this possibility is essential to inform the realistic application of personalized approaches in eating disorder care.

## 2 Method

### 2.1 Study Design

We conducted a secondary analysis of data collected as part of a randomized controlled non‐inferiority trial which compared a broad digital intervention, a focused digital intervention, and a waiting list for reducing eating disorder symptoms (Linardon et al. 2023). In the trial, participants were assessed at baseline, 4 weeks post randomization, and 8 weeks post randomization. Ethical approval was received from Deakin University and all participants provided informed consent.

### 2.2 Study Population and Recruitment

Participants were recruited in July–August 2021 via online advertisements posted on the senior author's psychoeducational eating disorder platform (https://breakbingeeating.com/) and its associated Instagram account (for more detail on this platform see Linardon et al. 2020). Interested participants were directed to complete an eligibility screening to ensure that they were: (1) over the age of 18 years, (2) had access to the Internet and a smartphone, and (3) self‐reported the presence of recurrent binge eating, defined as one episode per every 2 weeks, on average, over the past 3 months. All participants who met eligibility criteria were invited to complete the baseline assessment and were subsequently randomized into the broad intervention (*n* = 199), focused intervention (*n* = 199), or the waiting list (*n* = 202). The baseline assessment included self‐reported demographics, clinical history, and measures of symptom severity, motivation, confidence, and technology acceptance. The present study utilized data from participants randomized to the broad intervention or the focused intervention (*n* = 398).

### 2.3 Interventions

#### 2.3.1 Broad Intervention

The broad intervention, Break Binge Eating, was designed to target multiple hypothesized maintaining mechanisms of binge eating including dietary restraint, mood dysregulation, and body image concerns. The intervention was delivered through a smartphone application and consisted of four self‐paced modules (each 30–60 min to complete), with the first presenting psychoeducational material and the remaining three each focusing on one of the maintaining mechanisms. The content was based on Fairburn's transdiagnostic CBT protocol (Fairburn 2008) and was delivered via audio recordings, written text, and graphics within the app. The app also included a range of interactive features to enhance engagement including quizzes, a self‐monitoring diary, symptom tracking, and progress monitoring graphs.

#### 2.3.2 Focused Intervention

The focused intervention, Breaking the Diet Cycle, was a single target web program addressing dietary restraint. It consisted of four sessions (30–60 min each), one delivering psychoeducational content and three teaching skills related to self‐monitoring, adopting regular eating, and overcoming food anxiety. Content was based on Fairburn's transdiagnostic CBT protocol (Fairburn 2008) and was delivered through a web portal via written text, video tutorials, and graphics. Homework exercises, which encouraged participants to practice learned skills in their daily life, were completed through a smartphone app (e.g., completing a digital food diary).

Further details of the two interventions can be seen in the main outcome report (Linardon et al. 2023).

### 2.4 Measures

Given the fully remote design of the study and the large sample size, formal interview‐based diagnostic assessments were not feasible. The use of validated self‐report measures enabled standardized and scalable assessment of eating disorder psychopathology consistent with the trial design.

#### 2.4.1 Predictors

Forty predictors that were self‐reported at baseline were included. These consisted of demographics (i.e., age and gender), psychiatric diagnoses (i.e., current or past eating disorder diagnoses and co‐morbid psychiatric conditions), whether they were currently receiving treatment for eating disorder or body image issues, and their preference for either face to face treatment or a digital intervention. A range of eating disorder symptoms (i.e., dietary restraint, eating concerns, objective binge eating; OBE, subjective binge eating; SBE, compensatory behaviors, overvaluation, dissatisfaction, preoccupation, fear of weight gain, and feeling fat) were assessed via the Eating Disorder Examination Questionnaire (EDE‐Q; Fairburn et al. 2003). We also included several positive body image constructs including body image flexibility (Body Image Acceptance and Action Questionnaire‐5; BIAAQ‐5; Basarkod et al. 2018), body appreciation (Body Appreciation Scale‐2 Short Form; BAS‐2SF; Tylka and Wood‐Barcalow 2015), and functionality appreciation (Functionality Appreciation Scale; FAS; Alleva et al. 2017).

Other psychosocial/psychological factors included motivation to change unhealthy behaviors, motivation to use the digital program, confidence in ability to change, and depression and anxiety symptoms (Patient Health Questionnaire‐ 4; PHQ‐4; Kroenke et al. 2009). Digital intervention acceptance was also included, measured as the intent to use the technology as well as four factors influencing technology acceptance (i.e., performance expectancy, effort expectancy, social influence, and facilitating conditions) derived from the Unified Theory of Acceptance and Use of Technology (UTAUT; Venkatesh et al. 2003). Further detail on these measures and their rationale for inclusion is provided in the Supporting Informations.

#### 2.4.2 Outcome

Post‐treatment symptom severity was assessed via the EDE‐Q global score (Fairburn et al. 2003), which was self‐reported at 4 weeks post randomization and the primary outcome in the main trial. The global score was selected as it provides a validated and widely used index of overall eating disorder psychopathology, capturing cognitive, affective, and behavioral features within a single standardized metric and was pre‐registered as the primary endpoint of the trial.

### 2.5 Data Analysis

Statistical analyses were performed in R software (R Core Team 2021). A machine learning approach was used, adapted from the PAI framework proposed by DeRubeis et al. (2014). To predict individual‐level treatment response on each intervention, two machine learning algorithms were implemented: elastic net and random forest. Both models have been previously applied in PAI research within psychiatry, demonstrating promise (Delgadillo and Gonzalez Salas Duhne 2020; Meinke et al. 2024; Myers et al. 2024; Wade et al. 2025; Webb et al. 2022). Elastic net was chosen for its ability to perform feature selection, manage multicollinearity, and reduce overfitting by combining penalties of lasso (L1) and ridge (L2) regression (Zou and Hastie 2005). This makes it particularly suitable when the number of predictors is large relative to the sample size, or when predictors are likely to be correlated. However, elastic net is a linear model and requires interactions between variables to be included in the model a priori. Thus, we also tested a random forest algorithm which is capable of automatically detecting non‐linear relationships and higher order interactions. Random forest is an ensemble learning method that constructs multiple decision trees using bootstrapped samples of the training data (Cutler et al. 2012). At each decision point, the optimal split is determined from a random subset of predictors, and the model's final prediction is obtained by aggregating predictions across all trees (Cutler et al. 2012; Myers et al. 2024). Differences in performance between the two algorithms may indicate whether the relationship between chosen predictors and the outcomes is predominantly linear, and whether interactions between variables are present.

#### 2.5.1 Prediction Model Development and PAI Calculation

We conducted a complete case analysis (i.e., participants who provided post‐test outcome data), as multiple imputation was not feasible in the machine learning workflow. 184 participants had missing outcome data, resulting in a final sample of 214. There were no missing data on any predictor variables.

To develop and evaluate predictive models we used the caret (Kuhn, 2008), glmnet (Friedman et al. 2010; Tay et al. 2023) and randomForest (Liaw and Wiener 2002) packages. We employed 20 iterations to improve the stability of model performance, with identical seeds to ensure full reproducibility. Within each iteration, the full dataset was split (at random) into training (67%) and testing (33%) sets. 10‐fold cross‐validation was used inside each training set for hyperparameter selection and performance estimation (per recommendation by Meinke et al. 2024). All continuous predictors were centered and scaled (for elastic net model only as random forest is scale invariant).

Per prior recommendations, we trained separate models for each treatment by using treatment‐specific subsets of the training data (Meinke et al. 2024). This meant that the model predicting broad intervention outcomes was trained using only those who had received the broad intervention and the model predicting focused intervention outcomes was trained using those who had received the focused intervention. Predictive models were applied to generate factual predictions on the corresponding test subset (i.e., predicting the participants outcome on the treatment that they factually received), and counterfactual predictions by applying the alternative treatment's model to the same test data (i.e., predicting the participants outcome on the treatment they did not factually receive). To understand which variables were most influential for prediction, we obtained variable importance information for each model. Model coefficients were also extracted for the elastic net models.

In line with the PAI framework (DeRubeis et al. 2014), a PAI score was calculated for each participant in the test set by taking the difference between their predicted factual outcome and their predicted counterfactual outcome. In this case, a negative PAI indicates that the participant received their predicted optimal intervention (i.e., model predicted lower EDE‐Q global score in the factual treatment), while a positive PAI indicates a non‐optimal assignment (i.e., model predicted lower EDE‐Q global score in the counterfactual treatment). The absolute value of the PAI reflects the magnitude of the predicted treatment difference. For example, a PAI score of 1.5 indicates a predicted difference of 1.5 points in post intervention EDE‐Q global score between the two interventions.

#### 2.5.2 Evaluating Model Performance

Model performance was assessed by computing the root mean square error (RMSE), mean absolute error (MAE), and *R*‐squared (*R* ^2^) between the observed and predicted outcome scores in the test data. These metrics reflect the overall accuracy and explanatory power of the predictive models. For each iteration, predictions were generated using the treatment‐specific model corresponding to each participant's assigned condition, and the resulting prediction errors from the broad and focused models were combined to produce a single RMSE, MAE, and *R* ^2^ value per modeling approach (elastic net and random forest). Metrics from each iteration were then aggregated as a mean and standard deviation to summarize overall predictive performance. Individual level results across repeats were aggregated by participant, computing the mean PAI value and the modal recommended treatment across repetitions.

#### 2.5.3 Evaluating the Utility of the PAI

We evaluated the utility of PAI recommendations from the model (elastic net or random forest) with the highest predictive performance. Following previous methods (DeRubeis et al. 2014; Meinke et al. 2024; Wade et al. 2025), the utility of the PAI was evaluated by using an independent samples *t*‐test to compare post‐intervention EDE‐Q global scores between those who were randomized to their PAI recommended intervention versus those who were not. Cohen's *d* was used to quantify the magnitude of the effect. Prior to running *t*‐tests, we confirmed that EDE‐Q global scores were not significantly different between these two groups at baseline.

#### 2.5.4 Sensitivity Analysis

Similar to previous studies (e.g., Bauer‐Staeb et al. 2023; Delgadillo and Gonzalez Salas Duhne 2020; Tait et al. 2025), we repeated each *t*‐test in a subsample of participants with higher absolute PAI scores to investigate whether effects were stronger for participants with larger predicted treatment differences. Per prior studies, we defined a high PAI score as being greater than one standard deviation from the sample mean (Delgadillo and Gonzalez Salas Duhne 2020; Tait et al. 2025).

## 3 Results

### 3.1 Sample Characteristics

Sample characteristics are presented in Table S1. In the main trial, there were no group differences at baseline (Linardon et al. 2023).

### 3.2 Main Outcomes

Attrition analyses show that dropouts were younger (*d* = 0.19, *p* = 0.019), and reported more frequent subjective binge episodes (*d* = 0.17, *p* = 0.049) and compensatory behaviors (*d* = 0.19, *p* = 0.024) than completers. No other group differences emerged.

In the main trial, the mean EDE‐Q global scores at post‐intervention were 3.06 (SD = 1.14) for the broad group and 2.93 (SD = 1.27) for the focused group. There was no significant difference in the degree of change between the broad and focused interventions (*d* = −0.15, *p* = 0.280). However, both active interventions demonstrated moderate‐to‐large improvements relative to the control condition (between‐group effect sizes: broad vs. control *d* = −0.74; focused vs. control *d* = −0.89), indicating that the interventions were effective overall despite comparable group‐level outcomes.

### 3.3 Model Predictive Performance

Table 1 presents the mean predictive performance of the elastic net and random forest models for predicting post‐intervention outcome, over 20 iterations of test data sets (see Table S2 for treatment specific model performance). Overall, the average prediction error between factual and observed outcomes was comparable between models, with the predicted post‐intervention EDE‐Q global scores deviating from observed scores by approximately 0.8 points, on average. The elastic net model accounted for slightly more of the variance in observed outcomes (29%) compared to the random forest model (26%). Variable importance and coefficients for each model are reported in Tables S3 and S4.

**TABLE 1:** Mean Predictive Performance of Elastic Net and Random Forest Models for Predicting Post Intervention Outcome across 20 Iterations of Training and Testing Data Splits.

Modeling approach	RMSE	MAE	R^2^
Elastic net	0.99	0.81	0.29
Random forest	1.01	0.84	0.26

Abbreviations: MAE, mean absolute error; RMSE; root mean square error.

### 3.4 PAI Recommendations and Utility

The utility of the PAI generated from the elastic net model was evaluated given it performed marginally better compared to the random forest model. Across iterations, the mean absolute PAI score was 0.33 (SD = 0.12), indicating that, on average, participants were predicted to differ by 0.33 points on the EDE‐Q global score between their optimal and non‐optimal treatment condition (see Figure S1 for PAI distribution at the participant level). Table 2 presents the frequency with which each intervention was recommended, the proportion of participants who received their PAI recommended treatment, and the mean and standard deviation for post‐intervention EDE‐Q global scores across groups. As seen, the focused intervention was recommended more frequently than the broad intervention, and roughly half were randomized to their recommended treatment.

**TABLE 2:** Frequency of PAI Recommendations and Post Intervention Outcomes for Participants who were Randomized to their Recommended Intervention.

Model	Focused intervention recommended (*n*)	Broad intervention recommended (*n*)	Randomized to recommended intervention	*N*	Post‐intervention EDE‐Q global score *M* (SD)
Elastic net	137 (64%)	77 (36%)	Yes	99 (46%)	3.07 (1.30)
			No	115 (54%)	2.95 (1.10)

*Note:* Frequencies represent model‐recommended optimal treatment assignments (PAI) and do not reflect randomization ratios, which were equal across conditions.

An independent samples *t*‐test indicated no significant difference in observed post‐intervention EDE‐Q global score between those who did versus did not receive their PAI recommended intervention, *t*(193.7) = −0.73, *p* = 0.463, *d* = −0.10, 95% CI [−0.37, 0.17]. Baseline EDE‐Q global scores did not significantly differ between groups, *t(*205.88) = −1.71, *p* = 0.088, *d* = −0.24, 95% CI [−0.50, 0.03].

Although the mean post‐intervention EDE‐Q global score was numerically higher among participants randomized to their PAI‐recommended intervention, the difference was small (approximately 0.12 points) and not statistically significant. Critically, the PAI estimates the predicted difference in outcomes under each treatment for a given individual, rather than simply identifying those with lower symptom severity overall. In this sample, the mean absolute PAI was 0.33 points, indicating that the models generally predicted only modest between‐treatment advantages. When predicted differences are small, the expected benefit of matching is correspondingly limited, even in the presence of individual variability in outcomes.

### 3.5 Sensitivity Analyses

Independent samples *t*‐tests were repeated using a subsample of participants who had an absolute PAI score greater than one standard deviation from the sample mean, indicating a larger predicted treatment difference (28% of the sample, *n* = 60; See Figure S2 for distribution of the PAI). The mean absolute PAI in this sample was 0.59 (SD = 0.18). No significant difference was found in the mean observed post‐intervention outcome between those who received (*M* = 2.57) versus did not receive (*M* = 2.24) their PAI recommended intervention, *t*(43.20) = −0.99, *p* = 0.330, *d* = −0.26, 95% CI [−0.77, 0.25]. Baseline EDE‐Q global scores did not significantly differ between groups, *t*(49.67) = −1.38, *p* = 0.174, *d* = −0.36, 95% CI [−0.87, 0.15].

## 4 Discussion

This study aimed to investigate whether machine learning models could predict individual differences in treatment response to a broad versus focused digital intervention for eating disorder symptoms, and whether these predictions could be used to generate personalized treatment recommendations using the PAI framework (DeRubeis et al. 2014; Meinke et al. 2024). Overall, elastic net and random forest models achieved modest accuracy when predicting EDE‐Q global scores at post‐intervention, with predicted and observed outcomes differing by approximately 0.8 points, on average, across both models. Elastic net was selected as the preferred model due to obtaining a marginally lower prediction error and explaining a greater proportion of the variance in the outcome than the random forest. Although modest prediction accuracy was achieved, the predicted differences in post‐intervention outcomes between the two treatments were small (average difference of 0.3 points), and participants who received their PAI indicated optimal intervention did not show significantly better outcome than those who did not. This finding was also observed in the subsample of participants with a ‘high’ PAI (> 1 SD from the mean), representing those with larger predicted differences (28% of the sample), although the average PAI was modest even in this group (PAI *M* = 0.59). Together, these results indicate limited benefit of PAI based treatment matching for improving outcomes in this specific context.

These findings contrast with prior research in the broader mental health field, which has reported significant outcome improvements among individuals who received their PAI‐indicated optimal treatment (e.g., Deisenhofer et al. 2018; Delgadillo and Gonzalez Salas Duhne 2020). There may be several possible reasons for this discrepancy. First, it is possible that the baseline characteristics assessed primarily captured general predictors of symptom improvement, rather than true prescriptive predictors that differentiate who benefits more from one intervention versus the other. Although we included a broad range of theoretically relevant variables, these measures may not have been optimally aligned with the specific contrast between a broad versus focused CBT intervention, nor sufficiently sensitive to the mechanisms most likely to drive differential response. For example, more granular indices of the relative salience of mood intolerance versus dietary restraint, cognitive flexibility in engaging with multi‐component content, behavioral adherence patterns, or early response trajectories may have been more directly linked to the differential structure of the two programs. Relatedly, reliance on self‐reported baseline data may have limited measurement precision. Without strong, mechanism‐specific prescriptive predictors, the utility of the PAI is constrained (Cohen and DeRubeis 2018). This highlights a broader challenge within the field, as robust treatment moderators in digital eating disorder interventions remain poorly understood (McClure et al. 2024). Future research may benefit from incorporating more targeted process‐level measures, interview‐based assessments, or dynamic behavioral and digital data to better capture prescriptive signals.

Second, the potential for detecting differential effects may have been limited because the two interventions, while differing in scope and content, were both derived from the same therapeutic approach and delivered digitally. The original RCT was designed as a non‐inferiority trial, and we did not anticipate superiority of one program over the other at the group level. However, the purpose of the present study was not to detect mean differences, but to examine whether meaningful individual‐level heterogeneity in response could be identified despite equivalent average effects. The PAI framework assumes that subgroups of participants may respond differentially to credible, active treatments, even when overall effectiveness is comparable. Nevertheless, greater contrast in therapeutic modality or orientation may increase the likelihood of identifying prescriptive effects. Prior PAI studies (e.g., Huibers et al. 2015) have often compared interventions that differ more substantially in modality or approach, potentially providing greater opportunity for differential response patterns to emerge.

Third, smaller effects may reflect differences in analytical approaches. Specifically, the present study followed recent recommendations to minimize data leakage by applying preprocessing only on the training data and using 10‐fold cross validation instead of leave one out cross validation (LOOCV; Meinke et al. 2024). These more conservative procedures reduce the risk of overfitting and obtaining inflated estimates of predictive accuracy or treatment benefit. Many past PAI studies have relied on less stringent methods, which may have produced overly optimistic effects (Meinke et al. 2024). Thus, it is possible that methodological differences of this kind may also account for smaller PAI effects observed in the present study.

There are several limitations to consider when interpreting the results. First, the sample size was small for machine learning models, which can constrain model performance and reduce the likelihood of identifying underlying patterns which predict person‐specific treatment differences (Bzdok and Meyer‐Lindenberg 2018). Although there are no clear guidelines for minimum sample size requirements in machine learning, recommendations suggest including at least a few hundred observations per treatment arm to maximize the chances of detecting predictors of differential treatment response (Luedtke et al. 2019). Future research should prioritize collection of larger datasets or utilize existing sources of big data to optimize model performance.

A second limitation was that we were limited to self‐reported data which can contain a degree of measurement error, potentially reducing the precision of predictions (Berg et al. 2011). It may be worth exploring how interview‐based assessments or more novel data types (e.g., neuroimaging, smartphone sensor data) may be used to enhance prediction (Cohen and DeRubeis 2018).

A third limitation relates to the use of the EDE‐Q global score as the primary outcome. Although this is a validated and widely used measure of overall symptom severity, it does not fully capture behavioral symptoms, such as binge eating frequency. Given that study eligibility required engagement in binge eating, and that the interventions were designed to target behavioral symptoms, this may limit interpretation of treatment effects. Future research may benefit from including additional measures of eating disorder behaviors (e.g., Eating Pathology Symptoms Inventory; Forbush et al. 2013).

A fourth limitation was that the PAI was developed and evaluated using data from the same trial, which raises the possibility of circularity and inflated estimates of predictive performance. Although we sought to minimize overfitting by separating training and testing samples and employing internal cross‐validation, external validation in an independent dataset is required to establish generalisability. Further to this, because the PAI was applied retrospectively to existing trial data, it remains unknown whether prospectively using the PAI to allocate individuals to their predicted optimal treatment would yield superior outcomes. Demonstrating true clinical utility will require prospective trials in which treatment assignment is guided by PAI‐based recommendations. As this study represents a proof‐of‐concept and the first applications of the PAI to digital eating disorder interventions, external replication and prospective validation constitute important next steps.

In summary, our findings indicate limited utility of the PAI framework for matching individuals to different digital programs for eating disorder symptoms, as no significant differences in post‐intervention outcome were observed between those who did versus did not receive their model‐predicted optimal intervention. While it is possible that other variables may be needed to detect differential effects, findings likely suggest that, for most individuals, these interventions are similarly beneficial. Given the promise of PAI‐based treatment matching in other fields of research, it may be worthwhile to investigate whether greater benefits are observed when applied to two types of eating disorder treatments which differ in modality, delivery format, or therapeutic orientation. Advancing the ability to match individuals with their most suitable treatment option from the outset could have substantial benefits by enabling more efficient use of resources, reducing time spent on less effective treatments, and improving overall outcomes.

## Author Contributions

**Zoe McClure:** conceptualization, methodology, formal analysis, investigation, data curation, writing – original draft, writing – review and editing. **Matthew Fuller‐Tyszkiewicz:** investigation, writing – review and editing. **Mariel Messer:** investigation, writing – review and editing. **Jake Linardon:** conceptualization, investigation, writing – review and editing, data curation.

## Funding

The authors have nothing to report.

## Lived Experience Involvement Statement

Lived experience perspectives informed the design of the two intervention programs delivered in this secondary analysis.

## Conflicts of Interest

The authors declare no conflicts of interest.

## Supporting information


**Table S1:** Baseline Characteristics of Participants.
**Table S2:** Mean predictive performance of treatment specific models for predicting post intervention outcome across 20 iterations of training and testing data splits.
**Table S3:** Variable importance and coefficients for broad and focused elastic net models.
**Table S4:** Top 10 important predictors in the broad and focused random forest models.
**Figure S1:** Distribution of absolute PAIs at the participant level.
**Figure S2:** Distribution of absolute PAIs at the participant level for the high PAI subsample.

## Data Availability

The data that support the findings of this study are available from the corresponding author upon reasonable request.
