# Supplementary material for: Personalized Treatment Selection for Digital Eating Disorder Interventions: A Proof‐of‐Concept for the Personalized Advantage Index
Source: Int J Eat Disord. 2026 Apr 21;59(8):1785–93. doi: 10.1002/eat.70112 (PMC13446245; doi:10.1002/eat.70112)
Supplement: Supplementary file 1 [file EAT-59-1785-s001.docx]

**Supporting Information**

**Baseline Variables Included as Predictors in Models**

***Demographics***

Demographic predictors included participant age (in years) and identified gender.

***Psychiatric Diagnoses and Treatment***

Self-reported psychiatric diagnoses included past or current anorexia nervosa, bulimia nervosa, binge eating disorder, other specified feeding and eating disorder, major depressive disorder, or anxiety disorder. Participants also indicated whether they were currently receiving treatment for eating disorder or body image issues and their preference for either face to face treatment or a digital intervention.

These demographic and psychiatric diagnoses and treatment related predictors were selected as they are commonly collected in treatment efficacy trials and in clinical practice, making them practical and efficient to include in predictive models. Importantly, each provides insight into the participants’ baseline characteristics and clinical history, which may influence their response to an intervention.

***Eating Disorder Symptoms***

We included a range of eating disorder symptoms to investigate whether underlying symptom profiles influence predicted response to a broad program which targets several different maintaining mechanisms, compared to a focused program which specifically targets dietary restraint.

Eating disorder symptoms were assessed using subscale and item scores on the 18 item Eating Disorder Examination Questionnaire (EDE-Q; Fairburn et al., 2003). These included the global score, five-item eating restraint and eating concern subscales, the frequency of objective binge eating (OBE), and frequency of subjective binge eating (SBE) item. Responses on the self-induced vomiting, laxative use and driven exercise items were summed to form a composite item called compensatory behaviours. Several body image indicators, including shape/weight overvaluation (composite of two items), dissatisfaction (composite of two items), preoccupation (single item), fear of weight gain (single item), and feeling fat (single item) were also included as predictors (see Lydecker et al., 2017; Mitchison et al., 2017).

***Positive Body Image Constructs***

We included several positive body image constructs given that body image was a key maintaining mechanism targeted in the broad program. It is possible that those with less positive body image would benefit more from the broad program which addressed these concerns.

**Body Image Flexibility.** The Body Image Acceptance and Action Questionnaire- 5 (BIAAQ-5) was used to assesses body image flexibility (Basarkod et al., 2018). Five items are rated along a seven-point scale ranging from 1 (never true) to 7 (always true). Items are reverse scored and then summed to create a total score with higher scores reflecting a higher level of body image flexibility.

**Body Appreciation.** The Body Appreciation Scale-2 Short Form (BAS-2SF) was used to measure body appreciation (Tylka & Wood-Barcalow, 2015). Three items are rated along a five-point scale ranging from 1 (never) to 5 (always) and are averaged to create a total score. Higher scores reflect higher body appreciation.

**Functionality Appreciation.** The Functionality Appreciation Scale (FAS) was used to measure functionality appreciation (Alleva et al., 2017). Seven items are rated along a five-point scale ranging from 1 (strongly disagree) to 5 (strongly agree) and are averaged to create a total score. Higher scores reflect greater functionality appreciation.

***Other Psychological/Psychosocial Variables***

**Motivation to Change Unhealthy Behaviours.** Assessed using the item “How motivated are you to change any unhealthy eating behaviours (e.g., binge eating, yo-yo dietary)?”. Responses were rated on a scale from 0 to 10 with higher scores indicating greater motivation.

**Motivation to Use the Digital Program.** Assessed using the item “Right now, I am feeling motivated to use the digital mental health intervention I will receive”. Responses were rated on a scale from 0 to 10 with higher scores indicating greater motivation.

**Confidence in Ability to Change (Self-efficacy).** Assessed using the item “How confident are you in your ability to change any unhealthy eating behaviours (e.g., binge eating, yo-yo dieting)? Responses were rated on a scale from 0 to 10 with higher scores indicating greater confidence.

Motivation and confidence levels were assessed given past research has identified an association between these constructs and treatment outcomes (McClure et al., 2024). Additionally, it is possible that a focused program may be more suitable for those with lower motivation or confidence as a broad intervention may lead to premature disengagement if the content is not perceived as relevant.

**Depression and Anxiety Symptoms.** The four item Patient Health Questionnaire (PHQ-4; Kroenke et al., 2009) was used to assess symptoms of anxiety (two items) and depression (two items).

Depression and anxiety symptoms were included as a proxy for mood dysregulation which was a key maintaining mechanism targeted in the broad program. It is possible that those with broader mental health challenges benefit more from the broader program which addresses a wider range of concerns in comparison to the focused intervention which only targets dietary restraint.

***Digital Intervention Acceptance***

According to the Unified Theory of Acceptance and Use of Technology (UTAUT), intention to use the technology (i.e., acceptance) is a direct predictor of actual use (Venkatesh et al., 2003). Given links between engagement and treatment outcome (McClure et al., 2024), it is possible that acceptance may influence response to the digital intervention. Additionally, the main trial found that the focused intervention was associated with lower engagement, thus it is possible that those with lower acceptance may be more suited to the broader program (Linardon et al., 2023).

**Acceptance.** Consistent with prior work (Linardon et al., 2022), acceptance was assessed using four items derived from an existing measure of acceptance designed for digital interventions for depression (Ebert et al., 2015). Each item was slightly adapted to make it suitable for a digital intervention for eating disorder symptoms:

*“If I was exhibiting disordered eating symptoms, such as loss of control binge eating, intense body image concerns, restrictive eating, or driven exercise…”*

1. *I could imagine to try out a digital mental health intervention for these problems.*
2. *I would use a digital mental health intervention if offered.*
3. *I would recommend a digital mental health intervention to a friend.*
4. *I would be willing to pay for digital mental health intervention for these problems.*

Items were rated along a 5-point Likert scale with responses ranging from 1 (totally disagree) to 5 (totally agree). Total scores were derived by summing each item, where higher scores indicate greater acceptance. Past research has shown acceptable internal consistency for this measure (α = 0.75).

**Drivers of Acceptance.** We also included four drivers of acceptance (performance expectancy, effort expectancy, social influence, facilitating conditions), as informed by the UTAUT (Venkatesh et al., 2003). Items were adapted for use with eating disorder digital interventions as part of prior work (Linardon et al., 2022; see items below). Each item is rated along a 5-point Likert scale with responses ranging from 1 (does not apply at all) to 5 (applies completely). Items are summed to create a total score for each driver of acceptance. Past research has shown acceptable internal consistency for each subscale (performance expectancy, α = 0.85, Effort expectancy, α = 0.74; social influence, α = 0.68; facilitating conditions, α = 0.81).

***Performance Expectancy Items.*** *(1) Using a digital mental health intervention would reduce my disordered eating; (2) Using a digital mental health intervention for disordered eating would improve my personal well-being; (3) I would receive help for disordered eating from a digital mental health intervention; (4) Using a digital mental health intervention would help me to cope with disordered eating.*

***Effort Expectancy Items.*** *(1) Using a digital mental health intervention for disordered eating would cost me a lot of time and energy; (2) Using a digital mental health intervention would be an easy task for me; (3) A digital mental health intervention for disordered eating would be clear and easily comprehensible to me.*

***Social Influence.*** *(1) People close to me would recommend me to use a digital mental health intervention; (2) My general practitioner would recommend me to use a digital mental health intervention; (3) People close to me would use a digital mental health intervention in case they would suffer from disordered eating; (4) Other people would think badly about me if I would use a digital mental health intervention for disordered eating.*

***Facilitating Conditions.*** *(1) I have all the necessary technical preconditions for using a digital mental health intervention for disordered eating; (2) In case of technical problems with a digital mental health intervention, I would receive technical support; (3) I have the technical knowledge to utilize a digital mental health intervention*

| **Table S1**  *Baseline Characteristics of Participants* | |  |  |
| --- | --- | --- | --- |
| **Variable** | **Total sample**  **(n = 214)** | **PAI recommended broad (n = 77)** | **PAI recommended focused (n = 137)** |
| **Demographics** |  |  |  |
| Age (in years) | 34.8 (9.94) | 33.35 (8.8) | 35.61 (10.48) |
|  | **N (%)** |  |  |
| Gender |  |  |  |
| Female | 203 (95%) | 74 (96%) | 129 (94%) |
| Male | 11 (5%) | 3 (4%) | 8 (6%) |
| **Psychiatric diagnoses/treatment** |  |  |  |
| Anorexia nervosa |  |  |  |
| Past | 15 (7%) | 5 (7%) | 10 (7%) |
| Current | 1 (0.5%) | 1 (1%) | 0 (0%) |
| Bulimia nervosa |  |  |  |
| Past | 34 (16%) | 17 (22%) | 17 (12%) |
| Current | 16 (8%) | 10 (13%) | 6 (4%) |
| Binge eating disorder |  |  |  |
| Past | 62 (29%) | 28 (36%) | 34 (25%) |
| Current | 48 (22%) | 22 (29%) | 26 (19%) |
| Other specified feeding and eating disorder |  |  |  |
| Past | 8 (4%) | 3 (4%) | 5 (4%) |
| Current | 9 (4%) | 4 (5%) | 5 (4%) |
| Anxiety Disorder |  |  |  |
| Past | 98 (46%) | 43 (56%) | 55 (40%) |
| Current | 65 (30%) | 35 (46%) | 30 (21.9%) |
| Major Depressive Disorder |  |  |  |
| Past | 63 (30%) | 32(42%) | 31 (23%) |
| Current | 32 (15%) | 19 (25%) | 13 (10%) |
| Currently receiving treatment |  |  |  |
| Yes | 44 (21%) | 22 (29%) | 22 (16%) |
| No | 170 (79%) | 55 (71%) | 115 (84%) |
| Treatment preference |  |  |  |
| Face to Face | 39 (18%) | 13 (17%) | 26 (20%) |
| Digital Intervention | 175 (82%) | 64 (83%) | 111(80%) |
|  | **M (SD)** |  |  |
| **Eating disorder symptoms** |  |  |  |
| Preoccupation | 3.84 (214) | 5.03 (1.45) | 3.18 (2.18) |
| Overvaluation | 4.85 (1.52) | 5.74 (0.56) | 4.35 (1.66) |
| Dissatisfaction | 5 (1.37) | 5.56 (0.76) | 4.68 (1.52) |
| Fear of weight gain | 5.18 (1.55) | 5.88 (0.43) | 4.79 (1.8) |
| Feeling fat | 5.14 (1.57) | 5.79 (0.64) | 4.77 (1.8) |
| Compensatory behaviours | 12.16 (18.74) | 17.58 (23.3) | 9.12 (14.93) |
| Objective binge eating episodes | 16.27 (12.82) | 18.58 (14.56) | 14.96 (11.59) |
| Subjective binge eating episodes | 14.80 (16.62) | 18.44 (18.21) | 12.75 (15.34) |
| Eating restraint | 2.96 (1.46) | 3.64 (1.44) | 2.58 (1.34) |
| Eating concern | 3.58 (1.29) | 4.41 (0.86) | 3.12 (1.25) |
| Global | 3.89 (1.04) | 4.46 (0.56) | 3.46 (0.99) |
| **Positive body image** |  |  |  |
| Body image flexibility | 12.76 (5.99) | 8.12 (2.69) | 15.36 (5.75) |
| Body appreciation | 2.46 (0.70) | 2.18 (0.57) | 2.63 (0.71) |
| Functionality appreciation | 3.57 (0.76) | 3.23 (0.74) | 3.77 (0.7) |
| **Psychological/psychosocial** |  |  |  |
| Motivation to change | 8.75 (1.42) | 9.03 (1.21) | 8.6 (1.5) |
| Motivation to use | 8.82 (1.56) | 9.13 (1.16) | 8.64 (1.72) |
| Confidence | 5.50 (2.36) | 5.53 (2.35) | 5.47 (2.38) |
| Depression | 2.84 (1.91) | 3.57 (1.91) | 2.42 (1.79) |
| Anxiety | 3.29 (1.73) | 4.12 (1.51) | 2.83 (1.67) |
| **Technology acceptance** |  |  |  |
| Acceptance | 16.61 (2.12) | 17.18 (1.81) | 16.28 (2.22) |
| Performance expectancy | 15.57 (2.85) | 16.3 (2.4) | 15.15 (3) |
| Effort expectancy | 10.29 (2.11) | 10.68 (1.86) | 10.08 (2.22) |
| Social influence | 11.14 (3.36) | 11.71 (3.17) | 10.82 (3.44) |
| Facilitating conditions | 13.47 (2.35) | 14.01 (1.62) | 13.17 (2.63) |
|  |  |  |  |

| **Table S2**  *Mean Predictive Performance of Treatment Specific Models for Predicting Post Intervention Outcome across 20 Iterations of Training and Testing Data Splits* | | | |
| --- | --- | --- | --- |
|  | **RMSE** | **MAE** | **R^2^** |
| **Elastic Net** |  |  |  |
| Broad | 1.010 | 0.815 | 0.23 |
| Focused | 0.965 | 0.797 | 0.42 |
| **Random Forest** |  |  |  |
| Broad | 1.011 | 0.834 | 0.22 |
| Focused | 1.011 | 0.840 | 0.35 |
| Abbreviations: MAE, mean absolute error; RMSE; root mean square error | | | |

| **Table S3**  *Variable Importance and Coefficients for Broad and Focused Elastic Net Models* | | | | |
| --- | --- | --- | --- | --- |
| **Variable** | **Importance (broad model)** | **Coefficient**  **(broad model)** | **Importance (focused model)** | **Coefficient**  **(focused model)** |
| EDE-Q global | 100 | 0.37 | 100 | 0.53 |
| Dissatisfaction | 12.77 | 0.05 | 0 | 0 |
| Functionality appreciation | 1.59 | -0.01 | 5.71 | -0.03 |
| Body image flexibility | 0 | 0 | 28.25 | -0.15 |
| Body appreciation | 0 | 0 | 14.78 | -0.08 |
| Facilitating conditions | 0 | 0 | 12.26 | 0.06 |
| Eating concerns | 0 | 0 | 9.79 | 0.05 |
| Note. Predictors not included in the table did not contribute to either model. A zero coefficient does not indicate the predictor is unrelated to the outcome, rather that it does not contribute meaningfully above the other predictors, and thus its coefficient was shrunk to zero to avoid overfitting. | | | | |

| **Table S4**  *Top 10 Important Predictors in the Broad and Focused Random Forest Models* | | |
| --- | --- | --- |
| **Variable** | **Importance (broad model)** | **Importance (focused model)** |
| EDE-Q global | 100 | 100 |
| Eating concern | 76.95 | 63.06 |
| Body image flexibility | 73.03 | 52.35 |
| Eating restraint | 64.13 | 33.60 |
| Functionality appreciation | 57.58 | 21.77 |
| Dissatisfaction | 56.51 |  |
| Body appreciation | 51.23 | 21.67 |
| Overvaluation | 50.19 | 33.04 |
| Objective binge eating | 50.06 |  |
| Subjective binge eating | 49.07 |  |
| Preoccupation |  | 27.65 |
| Feeling fat |  | 27.49 |
| Social influence |  | 18.80 |
| Note. Importance score indicates how much the predictor contributes to improving the model’s predictions | | |

**Figure S1**

*Distribution of Absolute PAIs at the Participant Level*

Note. M = 0.27 (0.23), Min = 0.004, Max = 1.099

**Figure S2**

*Distribution of Absolute PAIs at the Participant Level for the High PAI Subsample*

Note, M = 0.59 (0.18), Min = 0.359, Max = 1.099

**References**

Alleva, J. M., Tylka, T. L., & Kroon Van Diest, A. M. (2017). The Functionality Appreciation Scale (FAS): Development and psychometric evaluation in U.S. community women and men. *Body Image*, *23*, 28-44. <https://doi.org/https://doi.org/10.1016/j.bodyim.2017.07.008>

Basarkod, G., Sahdra, B., & Ciarrochi, J. (2018). Body Image–Acceptance and Action Questionnaire–5: An Abbreviation Using Genetic Algorithms. *Behavior Therapy*, *49*(3), 388-402. <https://doi.org/https://doi.org/10.1016/j.beth.2017.09.006>

Fairburn, C. G., Cooper, Z., & Shafran, R. (2003). Cognitive behaviour therapy for eating disorders: a “transdiagnostic” theory and treatment. *Behaviour Research and Therapy*, *41*(5), 509-528. <https://doi.org/https://doi.org/10.1016/S0005-7967(02)00088-8>

Kroenke, K., Spitzer, R. L., Williams, J. B. W., & Löwe, B. (2009). An Ultra-Brief Screening Scale for Anxiety and Depression: The PHQ–4. *Psychosomatics*, *50*(6), 613-621. <https://doi.org/https://doi.org/10.1016/S0033-3182(09)70864-3>

Linardon, J., Anderson, C., Chapneviss, T., Hants, E., Shatte, A., & Fuller-Tyszkiewicz, M. (2022). Effects of an Acceptance-Facilitating Intervention on Acceptance and Usage of Digital Interventions for Binge Eating. *Psychiatric Services*, *73*(10), 1173-1176. <https://doi.org/10.1176/appi.ps.202100616> %M 35354324

Linardon, J., Shatte, A., McClure, Z., & Fuller-Tyszkiewicz, M. (2023). A broad v. focused digital intervention for recurrent binge eating: a randomized controlled non-inferiority trial. *Psychological Medicine*, *53*(10), 4580-4591. <https://doi.org/10.1017/S0033291722001477>

Lydecker, J. A., White, M. A., & Grilo, C. M. (2017). Form and formulation: Examining the distinctiveness of body image constructs in treatment-seeking patients with binge-eating disorder. *Journal of Consulting and Clinical Psychology*, *85*(11), 1095-1103. <https://doi.org/10.1037/ccp0000258>

McClure, Z., Fuller-Tyszkiewicz, M., Messer, M., & Linardon, J. (2024). Predictors, mediators, and moderators of response to digital interventions for eating disorders: A systematic review. *International Journal of Eating Disorders*, *57*(5), 1034-1048. <https://doi.org/https://doi.org/10.1002/eat.24078>

Mitchison, D., Hay, P., Griffiths, S., Murray, S. B., Bentley, C., Gratwick-Sarll, K., Harrison, C., & Mond, J. (2017). Disentangling body image: The relative associations of overvaluation, dissatisfaction, and preoccupation with psychological distress and eating disorder behaviors in male and female adolescents. *International Journal of Eating Disorders*, *50*(2), 118-126. <https://doi.org/https://doi.org/10.1002/eat.22592>

Tylka, T. L., & Wood-Barcalow, N. L. (2015). The Body Appreciation Scale-2: Item refinement and psychometric evaluation. *Body Image*, *12*, 53-67. <https://doi.org/https://doi.org/10.1016/j.bodyim.2014.09.006>

Venkatesh, V., Morris, M. G., Davis, G. B., & Davis, F. D. (2003). User Acceptance of Information Technology: Toward a Unified View. *MIS Quarterly*, *27*(3), 425-478. <https://doi.org/10.2307/30036540>
